# Temperature change exerts sex-specific effects on behavioural variation

**DOI:** 10.1098/rspb.2023.0110

**Published:** 2023-07-12

**Authors:** Jack A. Brand, Winston K. W. Yee, Ian J. Aitkenhead, Jake M. Martin, Giovanni Polverino, Steven L. Chown, Bob B. M. Wong, Damian K. Dowling

**Affiliations:** ^1^ School of Biological Sciences, Monash University, Melbourne, Victoria, 3800, Australia; ^2^ Department of Wildlife, Fish, and Environmental Studies, Swedish University of Agricultural Sciences, Umeå, Västerbotten, SE-907 36, Sweden; ^3^ Department of Zoology, Stockholm University, Stockholm, 10691, Sweden; ^4^ Centre for Evolutionary Biology, School of Biological Sciences, University of Western Australia, Perth, Western Australia, 6009, Australia; ^5^ Department of Ecological and Biological Sciences, Tuscia University, Viterbo, Lazio, 01100, Italy

**Keywords:** animal personality, behavioural plasticity, between-individual variation, climate change, physiology, within-individual variation

## Abstract

Temperature is a key factor mediating organismal fitness and has important consequences for species' ecology. While the mean effects of temperature on behaviour have been well-documented in ectotherms, how temperature alters behavioural variation among and within individuals, and whether this differs between the sexes, remains unclear. Such effects likely have ecological and evolutionary consequences, given that selection acts at the individual level. We investigated the effect of temperature on individual-level behavioural variation and metabolism in adult male and female *Drosophila melanogaster* (*n* = 129), by taking repeated measures of locomotor activity and metabolic rate at both a standard temperature (25°C) and a high temperature (28°C). Males were moderately more responsive in their mean activity levels to temperature change when compared to females. However, this was not true for either standard or active metabolic rate, where no sex differences in thermal metabolic plasticity were found. Furthermore, higher temperatures increased both among- and within-individual variation in male, but not female, locomotor activity. Given that behavioural variation can be critical to population persistence, we suggest that future studies test whether sex differences in the amount of behavioural variation expressed in response to temperature change may result in sex-specific vulnerabilities to a warming climate.

## 1. Introduction

Animals are confronted with a range of biotic and abiotic factors that vary across space and time. Temperature is one such factor, mediating organismal fitness, species distributions and ecological interactions [1–3]. Studies investigating how temperature affects organismal performance are especially important considering the role of human-induced climate change in driving increases in global temperatures and altering daily thermal variation [4–6]. These temperature changes are predicted to result in the local extinction of numerous species, posing a significant threat to biodiversity [7–9]. Understanding how species respond to thermal variation is, therefore, of great ecological importance.

Ectotherms are especially responsive to temperature changes due to their reliance on external heat to regulate internal body temperature and performance [2]. Much research has found that even transient temperature changes exert strong effects on the behaviour of ectotherms [2,10,11]. The thermal dependence of behavioural traits in ectothermic animals is thought to be due to the effect of temperature on underlying physiology, whereby higher temperatures (when experienced below thermal optima) increase metabolic rate and energy production [2,10]. As behaviour can determine organismal survival and reproductive success [12–14], the effect of temperature on behavioural and physiological traits may have important consequences for the ecological and evolutionary dynamics of populations.

However, recent research has revealed that within populations, not all individuals are similarly responsive to temperature change [15–19]. Importantly, individual differences in thermal behavioural plasticity may have broader consequences for animal populations by altering the amount of individual-level behavioural variation expressed across differing temperatures [18]. While relatively less is known about the role of temperature in mediating individual-level behavioural variance, recent work in a variety of ectotherms has found that rising temperatures can drive increases in behavioural variation both among- and within-individuals [17,18,20,21]. Here, it is thought that the positive relationship between temperature and metabolic rate in ectotherms results in a greater amount of energy available to express behavioural variation at higher temperatures [10,17]. These changes in individual-level variance may be key to the adaptive capacity of animal populations. Indeed, previous research has shown that increased among-individual behavioural variation increases colony fitness in ants (*Temnothorax longispinosus*; [22,23]). Similarly, while the ecological and evolutionary consequences of within-individual (i.e. residual) behavioural variation are not yet clear, prior studies have suggested that individuals may exhibit increased within-individual variation as an adaptive strategy for dealing with heightened predation risk [24,25]. Therefore, changes in individual behavioural variation in response to thermal fluctuations are expected to have implications for organismal fitness and population persistence in the face of environmental change.

Despite the importance of individual-level behavioural variance to population persistence, little is currently known about the intrinsic factors that mediate the effects of temperature on among- and within-individual behavioural variation. In particular, it is likely that such variation may differ across the sexes given the clear differences in life history between males and females, and the reported differences between males and females in their thermal responsiveness [26–28]. Indeed, a recent meta-analysis across 44 ectothermic species found a negative correlation between body mass and thermal acclimation capacity [27]. Interestingly, the mass-dependence of thermal acclimation capacity was associated with modest sex differences in thermal plasticity within species that exhibited sexual size dimorphism—potentially due to heavier organisms having greater thermal inertia in response to temperature change [27]. Moreover, males and females have also been shown to differ in their relationship between physiology and behaviour [29–31], suggesting that temperature-induced effects on physiological traits may exert sex-specific effects on behaviour. However, whether the effects of temperature on individual-level variation are sex-specific remains largely uninvestigated. Given that individual-level behavioural variation may promote population persistence in response to environmental change [32,33], sex differences in levels of thermally mediated among- and within-individual variation may have key ecological consequences.

Here, we investigated the potential for ecologically relevant temperature change to mediate sex-specific behavioural variation. Specifically, we tested the effects of temperature on individual-level variation in locomotor activity in male and female vinegar flies (*Drosophila melanogaster*), and explored whether any such effects are associated with underlying metabolic rates. The vinegar fly is a small ectothermic species that exhibits sex differences in both body size and energy management strategies [29,30], making it an ideal study subject. A recent meta-analysis found a negative association between body size and thermal acclimation capacity in ectotherms, whereby smaller size is associated with higher capacity for thermal acclimation [27]. Based on this reported association, we predicted that male flies would demonstrate increased population-level (i.e. mean-level) thermal plasticity in both their locomotor activity and metabolic rate due to their smaller body size in comparison to females, resulting in greater sex differences in activity and metabolic rate at 28°C compared to 25°C. We also aimed to identify whether temperature change altered both among- and within-individual variation in locomotor activity, and whether these effects differed between males and females. However, we had no clear predictions about how the effect of temperature on behavioural variance would differ between male and female *D. melanogaster*, and thus, we present these results as a first step in assessing sex differences in thermally mediated among- and within-individual variation in locomotor activity.

## 2. Methods

### (a) Fly collection and maintenance

The experimental population of *D. melanogaster* used in this study was originally collected from wild populations in Coffs Harbour, NSW, Australia. Details on the origin and maintenance of this population have been reported previously [34]. Briefly, 60 wild-caught non-virgin females were collected and transported to the animal facilities at Monash University. Offspring from each female (10 daughters and 10 sons) were mixed to create a single, mass-bred population. Flies were housed in standard vials (40 ml) on a potato dextrose–yeast–agar food medium (37.32% yeast, 31.91% dextrose, 23.40% potato medium and 7.45% agar combined with 98.48% water, 0.97% ethanol, 0.45% propionic acid and 0.11% nipagen) for approximately 260 discrete generations—corresponding to ten years of breeding—under standardized conditions (16 pairs per vial, across 10 vials, all adults admixed each generation prior to redistribution into individual vials; egg density limited to 100–120 per vial). Stocks were held within a controlled-temperature room (12 : 12 h light : dark cycle) maintained at 25°C (mean ± s.e. during experimental period = 24.57 ± 0.002°C), with the exception of unavoidable rare power outages or malfunctions of infrastructure (approx. three occasions over a decade) that led to short periods of thermal stress.

### (b) Experimental animals

Focal individuals (64 females and 65 males) were produced by parents and grandparents that were each 5 days of adult age at the time of egg-laying. Virgin male (body mass; mean ± s.e. = 0.66 ± 0.01 mg) and female (body mass; mean ± s.e. = 1.10 ± 0.01 mg) focal flies were collected under light CO_2_ anaesthesia within 6 h of eclosion. We chose to use only virgin flies as previous research has demonstrated significant effects of mating status on *D. melanogaster* locomotor activity and metabolic rate [30]. All flies were sorted into individual vials and left to recover for 3 days prior to the start of experiments. This 3-day recovery period ensured that any lasting effects of CO_2_ anaesthesia on fly physiology were eliminated by the time of experimental assays [35].

### (c) Behavioural experiments

Adult focal flies were 3 days post-eclosion at the start of behavioural trials. All flies were individually tested for locomotor activity across 6 seperate behavioural trials, each of 15 min in duration, over the course of 3 days. Behavioural assays were always performed within an approximately 2 h time window (13:49 – 15:35 h) to control for the previously reported variation in fly activity over the course of the day [30]. The locomotor activity of flies was tested at both their standard housing temperature (25°C) and at a high temperature (28°C). Data collected by the Australian Bureau of Meteorology from Coffs Harbour (where the original population was sourced) in 2020 indicate that average monthly minimum and maximum summertime temperatures range between approximately 19.7 – 29.2°C, with an approx. 7.3°C (± 0.07°C) average range in temperatures within a single day. Thus, 25°C and 28°C were chosen as they are within the thermal range that Australian *D. melanogaster* experience in the wild, and have been previously used to investigate thermal plasticity in this species [36].

At the beginning of behavioural trials, all flies were individually sorted into clean polycarbonate chambers (65 × 3 mm; length × width; volume = 0.46 ml) capped with 5 mm of foam at each end. Half of the flies underwent the first behavioural trial at their standard housing temperature (25°C), while the remaining individuals were first tested at the high temperature (28°C). Prior to the beginning of the trial, we measured the actual temperature of each individual polycarbonate chamber in both the 25°C (mean ± s.e. = 25.27 ± 0.02°C) and 28°C (mean ± s.e. = 27.53 ± 0.02°C) treatments using an infrared thermometer (Smart Sensor, Dongguan, China). All animals were given 60 min to acclimate to the testing temperature prior to the start of the assay. Behavioural trials were conducted in one of two separate assay chambers where fly activity was automatically tracked using ZebraLab software (ZebraBox, ViewPoint Behaviour Technology, Lyon, France). The temperature treatment of each assay chamber was randomized over the experiment to avoid any confounding effects of assay chamber on treatment temperature. Similar to previously established methods [37], we recorded the total distance that each fly moved (in mm) as a measure of locomotor activity over the 15 min trial.

After the completion of the first behavioural trial, flies were removed from the assay chamber and allowed to acclimate for 60 min to the test temperature of the second trial. This was set up so that those flies that were first tested at 25°C, were subsequently tested at 28°C, and *vice versa*. Following the conclusion of the second behavioural trial, flies were returned to their individual housing vials. This process was repeated each day for three days and allowed us to repeatedly measure the locomotor activity of each fly at both their standard housing temperature and at the high temperature. The order of the temperature treatments was alternated daily to control for any order effects. The experiment was run across four one-week sampling blocks that were each separated by one week; the focal flies used in each block were generated by independent sets of parental flies (*n* = 32 flies per block). The sex of flies and temperature treatment order were balanced within each block across the experiment to control for any differences between blocks.

### (d) Metabolic rate

After the completion of behavioural trials, all flies were tested for their standard (SMR) and active (AMR) metabolic rates at both their housing temperature (25°C) and a high temperature (28°C), following previously established prtocols [30]. Trials took place within one of two Panasonic MIR 352H-PE climate control cabinets (Panasonic Healthcare, Sakata, Japan) set at either 25°C (mean ± SE = 24.8 ± 0.01°C) or 28°C (mean ± SE = 28.2 ± 0.02°C). Metabolic trials were conducted for 9.5 h overnight (21:00 – 06:30) across two separate nights (see electronic supplementary material for detailed metabolic rate methods). Flies that underwent the first metabolic rate trial at 25°C were subsequently tested at 28°C during the second trial in a distinct metabolic rate chamber, and *vice versa* for flies initially tested at 28°C. Similar to behavioural trials, temperature treatment order was fully balanced across sexes. We measured the rate of CO_2_ production (VCO_2_ µl h^−1^) of each fly as a proxy for metabolic rate using eight Sable Systems International (SSI, Las Vegas, Nevada, USA) multiple animal versatile energetics systems (MAVEn), each attached to a Li-Cor 7000 CO_2_/H_2_O infrared gas analyser (Li-Cor, Lincoln, Nebraska, USA).

Flies were individually sorted without anesthesia into clean polycarbonate chambers (65 × 3 mm; volume = 0.46 ml) capped with 5 mm of foam at each end. Four randomly chosen individuals (2 males and 2 females) were then gently loaded into one of eight MAVEn systems where they remained until the end of the trial. Individual chambers were sequentially measured for a period of 10 min each, with a baseline recording (5 min) taken between each measurement to account for drift in the Li-Cor 7000 throughout the experiment. This was repeated six times over the course of each trial, resulting in six VCO_2_ measurements for each individual fly at both 25°C and 28°C. Flow rate was set by the MAVEn system and held constant at 15 ml min^−1^ throughout all experiments. We also simultaneously recorded the routine movement of each fly during each measurement using infrared light detectors in the MAVEn activity board. Movement was detected through changes in the infrared light field above each detector and is presented as a unitless measurement corrected to an absolute difference sum (ADS-movement). Specifically, ADS-movement is calculated by sequentially adding the absolute differences between adjacent data points from deflections in the infrared light detectors above each metabolic rate chamber. While ADS-movement is not an absolute measure of locomotion, it can be likened to the ‘intensity’ of movement exhibited by the animal and is widely used in the literature to account for variance in metabolic rate due to variation in organismal activity during the recording [38,39]. For each fly, we extracted the mean VCO_2_ from each 10 min recording, as well as the corresponding range in ADS-movement. Similar to previous research [40], we took the recording period with the lowest and highest VCO_2_ readings at both 25°C and 28°C as measures of SMR and AMR, respectively.

Following the completion of the metabolic rate assay, we measured the body mass of each fly using a fine micro-balance (±0.0001 mg; Cubis series MSA2.7s-000-DM microbalance, Sartorius AG, Goettingen, Germany).

### (e) Statistical analysis

Data were analysed using *R* v. 4.0.3 [41]. A total of 768 behavioural trials (i.e. 192 h of behavioural recording) from 129 individuals (64 females and 65 males) were included in the analysis. One male escaped after the first day of behavioural recordings and was replaced by a male conspecific of the same age from the same block. All continuous covariates were mean-centred and scaled (mean = 0; s.d. = 1), while the chamber in which behavioural trials were conducted in (1 or 2) was centred (i.e. chamber 1 = –0.5; chamber 2 = 0.5) prior to analysis to aid in model fitting and interpretation (see [42]). In all analyses, we used the *brms* package [43] to fit Bayesian linear mixed-effects models to investigate sex differences in locomotor activity and metabolic rate. All models were run for 5000 iterations (1000 warmup), with a thinning interval of two, and on four chains using relatively uninformative, default priors. Model convergence was visually checked via trace plots, with all chains converging $\left(\hat{R}=1\right)$. Inference was based on posterior means and their associated 95% credible intervals (CI).

We first used a Bayesian, double-hierarchical generalized linear mixed-effects model to investigate sex differences in behavioural variation across the two different temperatures (table 1). Briefly, this approach allows the explicit modelling of both mean (i.e. mean-model) and residual (i.e. residual model) level behavioural variation within a single overarching framework [44]. Preliminary analysis found no substantial effect of either experimental block (*F*_3,122_ = 0.05, *p* = 0.99) or temperature treatment order (*F*_1,123_ = 1.47, *p* = 0.23) on locomotor activity, and therefore, these variables were excluded from the final model to reduce model complexity. For the final double-hierarchical model, we included total distance moved (mm) as the response variable, while the mean model included body mass (mg), trial number (1–6), assay chamber (1 or 2) and time of day (13 : 49–15 : 35 h; coded as min since 13 : 00 h) as covariates, while sex (male or female) and temperature (25°C versus 28°C) were included as fixed-effect factors. The final model also included a sex by temperature interaction. To test for sex differences in behavioural variation, we fitted individual ID as a random intercept separately for each sex and allowed this to differ between the two temperature treatments. In the residual model, we allowed variance estimates to differ between males and females at each temperature to investigate how sexes differed in their residual, within-individual behavioural variation across temperature treatments. A recent meta-analysis found that individuals often differ from one another in their within-individual behavioural variance [45]. While not the focus of the current study, we nevertheless included individual ID as a random intercept in the residual model to control for any among-individual differences in within-individual variance. Following model fitting, we extracted all variance estimates and calculated the magnitude difference in among-individual (i.e. ΔV_I_) and residual within-individual (i.e. ΔV_W_) variance between treatment groups to statistically compare how males and females differed in the effect of temperature on behavioural variation (e.g. [46–48]). Similarly, we calculated the coefficient of both among-individual (CV_I_) and within-individual (CV_W_) variation in locomotor activity for males and females at both temperature treatments. The coefficient of variation is a mean-standardized variance estimate that disentangles the effect of temperature change on behavioural variation from mean-level changes in locomotor activity [49,50]. As above, we took the magnitude difference in coefficients of among- (ΔCV_I_) and within-individual (ΔCV_W_) variation to statistically compare treatment groups. We also report adjusted repeatability estimates here for both sexes at both temperature treatments for completeness (table 2).

**Table 1.** Model estimates (± 95% CI) extracted from the Bayesian linear mixed-effects model investigating sex differences in locomotor activity. Estimates are given for both the mean model (i.e. average behaviour) and the residual model (i.e. within-individual behavioural variation). Fixed-effects estimates from the mean model displayed in bold are those whose CI do not overlap zero (note: variance estimates cannot overlap with zero as they are positively bound). Females at 25°C are set as the reference group. Variance estimates from the mean and residual model were converted back to the original scale for each treatment group from brms model output.

model	variable	estimate (± 95% CI)
**mean model**	*fixed-effects*	
	intercept	**2009.98 (1844.40, 2182.24)**
	sex	
	male	**703.38 (389.34, 1021.03)**
	temperature	
	28°C	**152.13 (70.55, 233.68)**
	body mass (mg)	–92.77 (–244.69, 57.60)
	trial number	**–29.10 (–56.64, –1.83)**
	assay chamber	**84.04 (31.06, 136.34)**
	time of day	**34.68 (5.72, 63.80)**
	sex:temperature	124.12 (–2.37, 249.19)
	*random-effects*	
	male V_I_	
	individual ID – 25°C	150 346 (81 236, 222 662)
	individual ID – 28°C	260 187 (149 726, 384 940)
	cor(individual ID 25°C, 28°C)	0.94 (0.83, 1.00)
	female V_I_	
	individual ID – 25°C	97 728 (44 402, 154 258)
	individual ID – 28°C	96 831 (56 531, 142 864)
	cor(Individual ID 25°C, 28°C)	0.90 (0.72, 1.00)
**residual model**	*fixed-effects*	
	male V_W_	
	25°C	170 220 (97 430, 244 196)
	28°C	285 181 (169 032, 417 673)
	female V_W_	
	25°C	163 669 (118 959, 209 286)
	28°C	79 008 (57 728, 100 576)
	*random-effects*	
	male – individual ID	4.05 (2.95, 5.33)
	female – individual ID	1.53 (1.07, 1.95)

**Table 2.** Coefficients of among- (CV_I_) and within-individual (CV_W_) variation and adjusted repeatability estimates (±95% CI) for the locomotor activity of females and males at both 25°C and 28°C.

		CV_I_	CV_W_	repeatability
sex	temp	estimate (± 95% CI)	estimate (± 95% CI)	estimate (± 95% CI)
female	28°C	0.14 (0.11, 0.18)	0.13 (0.11, 0.15)	0.55 (0.41, 0.68)
	25°C	0.15 (0.11, 0.20)	0.20 (0.17, 0.23)	0.37 (0.21, 0.52)
male	28°C	0.17 (0.13, 0.21)	0.18 (0.14, 0.22)	0.48 (0.31, 0.63)
	25°C	0.14 (0.11, 0.18)	0.15 (0.12, 0.19)	0.47 (0.31, 0.63)

We ran two univariate generalized linear mixed-effects models to investigate sex differences in the population-level thermal plasticity of metabolic rate (see electronic supplementary material, tables S2 and S3 for full model output). Some individuals were lost due to early mortality prior to the completion of metabolic rate trials, resulting in a total of 64 females and 57 males that completed all metabolic rate trials and were included in the model. Routine movement data of flies during the metabolic rate trials (ADS-movement) was log_10_ + 1 transformed prior to analysis. Both SMR and AMR were each included as the respective response variables in two separate models, while body mass, relative humidity (95.39–97.67%), trial day (day 1 versus day 2), and ADS-movement of each individual fly during the trial were included as covariates. In addition, we included sex and temperature (25°C versus 28°C) as fixed-effects factors, as well as interactions between sex and mass, sex and temperature, and sex and ADS-movement in the model. Individual ID was included as a random intercept separately for each sex. In these models, a significant interaction between sex and temperature would indicate that males and females differed in how they altered their metabolic rate across the temperatures (i.e. sex differences in population-level thermal metabolic plasticity).

We found that males and females marginally differed from each other in their population-level thermal plasticity in locomotor activity (see §3). Given that sex differences in body size may contribute to variation between males and females in their thermal plasticity [27], we also ran a *post-hoc* analysis to investigate whether the sex by temperature interaction in the mean locomotor activity model ‘disappeared’ when conditioning on a body mass by temperature treatment interaction (i.e. suggesting that body mass differences between males and females may be driving sex differences in thermal plasticity). The model structure was identical to the locomotor activity analysis described above, with the addition of a body mass by temperature interaction in the mean model. As males and females did not differ in their population-level metabolic plasticity (see §3), we did not include a post-hoc analysis for the SMR or AMR models.

Finally, as previous research has found genetic correlations between locomotor activity and metabolic rate in male, but not in female *D. melanogaster* [29], we also ran two bivariate generalized linear mixed-effects models to investigate potential sex differences in the relationship between locomotor activity and metabolic rate (both SMR and AMR). Both activity and either SMR or AMR (depending on the model) were included as the response variables. The activity, SMR, and AMR models contained the same fixed-effects as described directly above. Individual ID was included as a random intercept separately for each sex in all models. We estimated sex-specific among-individual correlations between locomotor activity and either SMR or AMR, respectively (note that correlations were not temperature-treatment specific as we did not have repeated measures of SMR or AMR at either 25°C or 28°C). ADS-movement was retained as a covariate in both the SMR and AMR models to control for the effect of routine movement on metabolism, following previously established methods [29,30]. However, for completeness, we also ran a supplementary analysis where metabolic rate was not corrected for ADS-movement during the trial. The correlation estimates between locomotor activity and either SMR or AMR uncorrected for ADS-movement were qualitiatively similar to those reported in the main text (see electronic supplementary material).

## 3. Results

### (a) Mean-level effects: locomotor activity

**Figure 1.**
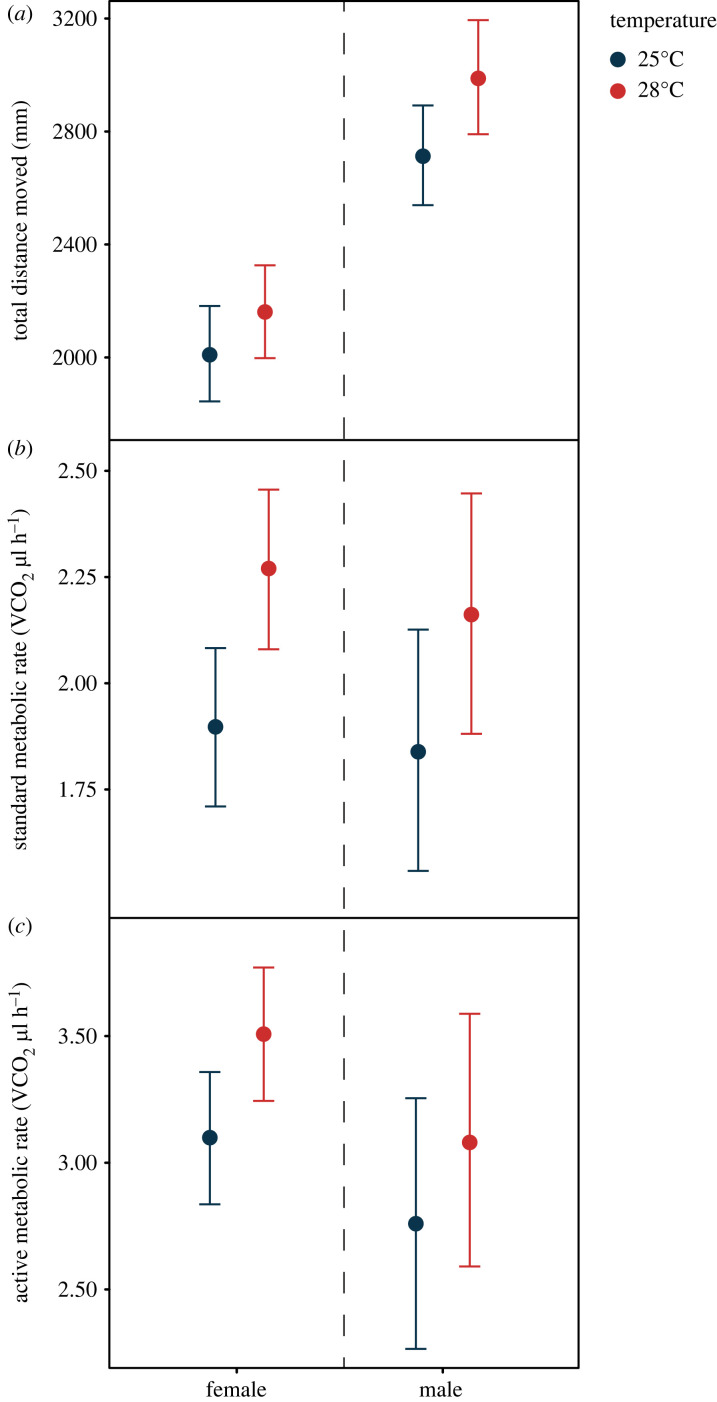
Population-level thermal plasticity of (*a*) locomotor activity, (*b*) standard metabolic rate (SMR), and (*c*) active metabolic rate (AMR) in both males and females. Plots represent conditional effects (± 95% CI) extracted from Bayesian linear mixed-effects models for both females (activity: *n* = 64; metabolic rate: *n* = 64) and males (activity: *n* = 65; metabolic rate: *n* = 57), with estimates displayed in blue and red for assays conducted at 25°C and 28°C, respectively.

Males and females marginally differed from each other in their population-level thermal behavioural plasticity (i.e. sex × temperature interaction; figure 1*a*; table 1 & electronic supplementary material, table S1). While both sexes increased their locomotor activity in response to the high-temperature treatment (female estimate [95% CI] = 152.13 [70.55, 233.68]; male estimate [95% CI] = 276.25 [177.93, 374.44]), males displayed a moderately greater increase in their activity in response to increased temperature when compared to females (table 1). However, this effect was only partly supported with CI overlapping zero (table 1). When including a body mass by temperature interaction in the *post-hoc* model, there was no longer any interaction between sex and temperature on locomotor activity (sex × temperature interaction in the *post-hoc* model [95% CI] = –5.08 [–283.18, 284.64]), suggesting that the marginal sex difference in population-level behavioural thermal plasticity was likely driven by differences in body mass between males and females. Males were also more active than females at both temperature treatments, after controlling for sex differences in body mass (figure 1*a*; table 1). Furthermore, trial number, time of day and assay chamber all had an effect on locomotor activity. However, while there was a marginally negative effect of body mass on locomotor activity, CIs for this effect were wide and included zero (table 1).

### (b) Among- and within-individual variation: locomotor activity

**Figure 2.**
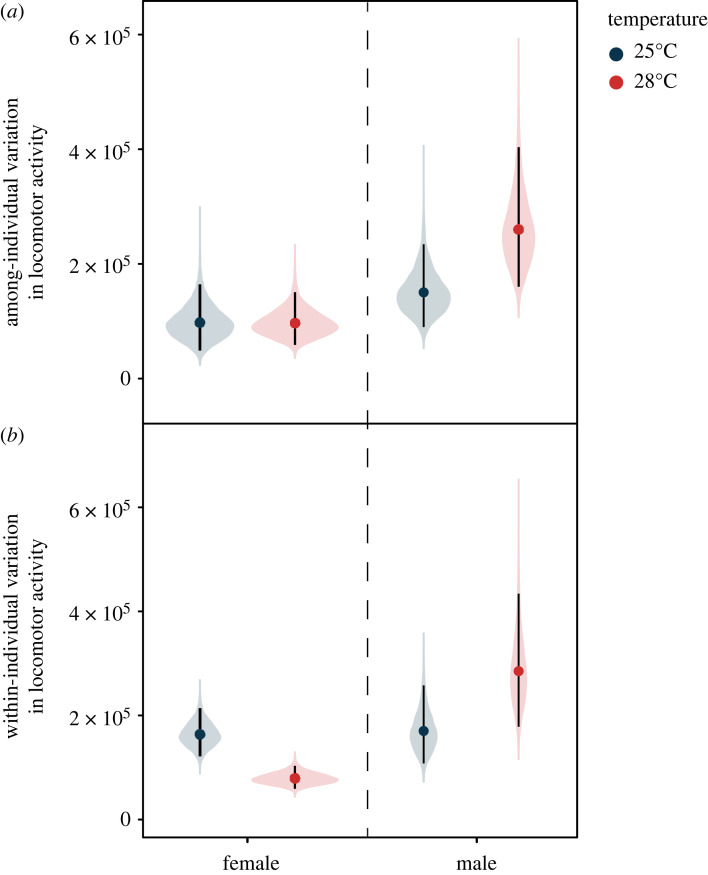
Sex differences in the effect of temperature on (*a*) among-individual and (*b*) within-individual variation in locomotor activity. Filled circles represent mean variance estimates for both males and females tested at 25°C (blue) and 28°C (red), respectively, while vertical error bars denote 95% CI, and plot width represents the probability density.

Among-individual variation in locomotor activity increased in males in response to higher temperature (ΔV_I_ = 109 840 [14 496, 209 594]; figure 2*a*; table 1). By contrast, in females there was no difference in among-individual behavioural variation across the temperature treatments (ΔV_I_ = –897 [–59 146, 53 792]; figure 2*a*; table 1). This sex difference in variance across the temperature treatments resulted in greater among-individual behavioural variation in males, when compared to females at 28°C (ΔV_I_ = 163 355 [39 082, 294 567]), but not at 25°C (ΔV_I_ = 52 617 [–40 960, 146 979]). Furthermore, both males and females demonstrated positive among-individual correlations in their activity across the temperature treatments that were close to 1 (table 1), suggesting that the rank-order of among-individual differences in locomotor activity was maintained across the temperature treatments in both sexes [51].

However, coefficients of among-individual variation differed little between the sexes across the temperature treatments, suggesting that the sex differences in the effect of temperature on among-individual variation were largely driven by changes in mean-level locomotor activity. Neither males (ΔCV_I_ = 0.03 [–0.01, 0.06]; table 2) nor females (ΔCV_I_ = –0.01 [–0.06, 0.03]; table 2) substantially altered their CV_I_ across the temperature treatments. Similarly, there were minimal sex differences in CV_I_ at both 25°C (ΔCV_I_ = –0.01 [–0.07, 0.05]: table 2) and 28°C (ΔCV_I_ = 0.03 [–0.03, 0.08]; table 2).

Temperature exerted sex-specific effects on within-individual behavioural variation (figure 2*b*; table 1). While within-individual variation in activity increased for males in response to higher temperatures (ΔV_W_ = 114 961 [22 940, 223 542]; figure 2*b*; table 1), the opposite pattern was observed for females, which displayed lower levels of within-individual variance when tested at higher temperatures (ΔV_W_ = –84 661 [–131 768, –38 671]; figure 2*b*; table 1). Thus, while there was no substantial difference between males and females in their within-individual behavioural variation at 25°C (ΔV_W_ = 6550 [–80 626, 95 534]; table 1), this changed when flies were tested at 28°C, with males displaying substantially greater within-individual behavioural variation when compared to females (ΔV_W_ = 206 173 [84 443, 334 899]; table 1).

Furthermore, there was little evidence for a substantial increase in the coefficient of within-individual variation in males at higher temperature (ΔCV_W_ = 0.03 [–0.01, 0.06]; table 2). However, females substantially reduced their coefficient of within-individual variation at higher temperatures (ΔCV_W_ = –0.07 [–0.10, –0.04]; table 2), suggesting that the effect of temperature on within-individual variance in females was distinct from mean-level effects. While males demonstrated a marginally reduced coefficient of within-individual variation at 25°C compared to females (ΔCV_W_ = –0.05 [–0.10, 0.00]; table 2), this sex difference was reversed at 28°C, where males displayed an increased coefficient of within-individual variation relative to female conspecifics (ΔCV_W_ = 0.05 [0.00, 0.09]; table 2).

### (c) Metabolic rate

SMR increased at 28°C in both males (estimate [95% CI] = 0.32 [0.20, 0.45]) and females (estimate [95% CI] = 0.37 [0.25, 0.49]) when compared to their SMR at 25°C (figure 1*b*; electronic supplementary material, table S1). However, there were no sex differences in the population-level thermal plasticity of SMR (i.e. no sex × temperature interaction; estimate [95% CI] = –0.05 [–0.22, 0.12]; figure 1*b*). Males and females also did not differ in their SMR at either 25°C (estimate [95% CI] = 0.06 [–0.29, 0.40]) or 28°C (estimate [95% CI] = 0.11 [–0.22, 0.44]). Moreover, SMR was positively related to ADS-movement during the metabolic rate trial in both males (estimate [95% CI] = 0.28 [0.20, 0.35]) and females (estimate [95% CI] = 0.14 [0.05, 0.24]). However, males displayed substantially higher metabolic rates with increasing ADS-movement during the trial compared to females (sex × ADS-movement interaction; estimate [95% CI] = 0.13 [0.01, 0.25]; electronic supplementary material, figure S1*a*). As expected, body mass had a positive effect on SMR (female estimate [95% CI] = 0.29 [0.10, 0.47]; male estimate [95% CI] = 0.26 [–0.01, 0.54]). While there was increased uncertainty around the effect of mass on SMR in males (CIs slightly overlapping with zero), the relationship between mass and SMR did not statistically differ between sexes (i.e. no sex × body mass interaction; estimate [95% CI] = –0.03 [–0.36, 0.31]). Furthermore, flies marginally increased their SMR in response to increased relative humdity (estimate [95% CI] = 0.04 [–0.01, 0.09]) and marginally decreased their SMR over repeated trials (estimate [95% CI] = –0.04 [–0.09, 0.00]). However, these effects were relatively small, with CIs slightly overlapping with zero.

Similar to SMR, AMR also increased at 28°C in both males (estimate [95% CI] = 0.32 [0.12, 0.53]) and females (estimate [95% CI] = 0.41 [0.21, 0.62]) when compared to their AMR measured at 25°C (figure 1*c*; electronic supplementary material, table S1). There were no sex differences in the population-level thermal plasticity of AMR (i.e. no sex × temperature interaction; estimate [95% CI] = –0.09 [–0.38, 0.21]; figure 1*c*). Moreover, males and females did not differ substantially in their AMR at either 25°C (estimate [95% CI] = 0.34 [–0.25, 0.89]) or 28°C (estimate [95% CI] = 0.43 [–0.15, 0.99]). However, the sexes differed in their relationship between AMR and ADS-movement during the trial (i.e. sex × ADS-movement interaction; estimate [95% CI] = 0.30 [0.12, 0.49]; electronic supplementary material, figure S1*b*), whereby there was a positive relationship between AMR and movement in males (estimate [95% CI] = 0.31 [0.16, 0.45]) but not in females (estimate [95% CI] = 0.01 [–0.11, 0.12]). While flies decreased their AMR over repeated trials (estimate [95% CI] = –0.17 [–0.24, –0.09]), we found no effect of relative humidity during the trial, body mass or a sex by body mass interaction on their AMR (i.e. all CIs overlapped with zero).

### (d) Among-individual correlation between locomotor activity and metabolic rate

We found no clear evidence for among-individual relationships between locomotor activity and metabolic rate in either sex. While correlations between locomotor activity and SMR were in opposite directions for males (*r* [95% CI] = 0.23 [–0.85, 0.96]) and females (*r* [95% CI] = –0.42 [–0.95, 0.42]), there was substantial uncertainty around both estimates, with CI overlapping with zero. This was similar for the correlation between locomotor activity and AMR in both males (*r* [95% CI] = 0.51 [–0.29, 0.97]) and females (*r* [95% CI] = –0.07 [–0.91, 0.87]), providing no clear evidence for relationships between locomotor activity (from behavioural experiments) and metabolic rate (both corrected and uncorrected for ADS-movement during the trial; see electronic supplementary material) at the among-individual level in either sex.

## 4. Discussion

We predicted that sexual dimorphism in the body size of *D. melanogaster* would result in males displaying, on average, increased population-level thermal plasticity in their locomotor activity and metabolic rate, when compared to females. In line with our predictions, we found increased population-level plasticity in locomotor activity in response to temperature change in males relative to females. However, this was not the case for either standard (SMR) or active (AMR) metabolic rate, with both males and females similarly increasing their metabolism in response to higher temperatures. In addition, we found evidence that temperature change exerts sex-specific effects on both among- and within-individual variation in locomotor activity. These results may have possible implications for population persistence in the face of environmental change, which we discuss below.

We found that males increased their locomotor activity to a greater extent than females in response to rising temperatures, and that this effect was likely attributable to sex differences in body size. However, we should note that there was uncertainty around this effect, with credible intervals partially overlapping zero. Nevertheless, previous work has also demonstrated sex differences in the population-level thermal behavioural plasticity of *D. melanogaster* [36,52]. In particular, while the average activity of males has been shown to increase with higher temperatures, female activity rates plateaued at temperature increases above 24°C [52]. Similarly, previous research found that male *D. serrata* maintained higher activity rates across a broader range of temperatures than females, resulting in wider thermal performance curves in males than females [26]. These sex differences in the thermal plasticity of locomotor activity may have substantial fitness consequences. Indeed, prior research has suggested that locomotor activity may be under sexually antagonistic selection in *D. melanogaster*, whereby increased activity rates result in high reproductive fitness in males, but decreased fitness in females [53]. Therefore, the greater increase in activity levels observed in males, relative to females, in response to high temperatures may be an adaptive response to maximize fitness under contrasting thermal environments. Future research testing locomotor activity and fitness across a broader range of temperatures will be needed to investigate whether the sex differences in population-level thermal plasticity are adaptive. Previous work in *D. melanogaster* has demonstrated that mating status, starvation, and the social context of flies during the assay may influence locomotor activity, and that such effects may differ between the sexes [30,37,54]. We used satiated virgin flies that were tested in an asocial context; whether the marginal sex difference in the thermal plasticity of locomotor activity is maintained in mated flies tested across different levels of food deprivation and varying social conditions is not clear and will require further research.

Despite our results showing sex differences in thermal behavioural responsiveness, we found no substantial sex differences in the population-level thermal plasticity of either SMR or AMR. More specifically, contrary to our predictions that the smaller body size of males would result in greater population-level thermal metabolic plasticity, we found that both male and female flies were similarly responsive in their SMR and AMR to rising temperatures. Previous research in *D. melanogaster* also found no evidence for greater population-level thermal metabolic plasticity in males compared to females [36]. Indeed, males flies were actually shown to be less responsive in their metabolic rate to temperature change than females [36]. Taken together, this suggests that the reduced body size of male *D. melanogaster*, relative to females, does not result in males being more metabolically plastic, on average, in response to changes in the thermal environment. Why sexes differed in their thermal behavioural, but not in metabolic plasticity, at the population vel remains unclear. We surmise that this may have been due to sex differences in energy management strategies. For example, we found that the relationship between activity and either SMR or AMR was in opposite directions for males and females, albeit with substantial uncertainty around these estimates (CIs overlapping with zero). Indeed, previous research has reported a positive genetic correlation between locomotor activity and SMR in *D. melanogaster* males, but not females [29]. Thus, potential sex differences in the relationship between locomotor performance and metabolism may explain the current findings, whereby equal increases in SMR at higher temperatures are associated with increased activity rates in males, but not females. However, whether there are sex differences in genetic covariance between locomotor activity and SMR in our study population, and whether these genetic covariances change across different temperature treatments is not known and would therefore benefit from future quantitative genetic and metabolomic studies (e.g. [55,56]).

We also found that temperature altered among-individual variation in locomotor activity, and that this effect was sex-dependent. Further, male and females flies demonstrated positive among-individual correlations in their locomotor activity across the temperature treatments that were close to 1, suggesting that the rank order of among-individual differences was maintained across the temperature treatments. Previous work in ectotherms has found that individuals differ from each other in their behavioural response to temperature change, resulting in changes to among-individual variance [17,18]. However, whether patterns of temperature-dependent among-individual behavioural variance may differ between the sexes has previously been overlooked. While among-individual variation in locomotor activity increased at higher temperatures in males, this was not the case for females, which did not differ in their among-individual variation across the temperature treatments. This resulted in males showing greater among-individual variation compared to females at 28°C, but not at 25°C. Previous research has reported greater additive genetic variance for locomotor activity in male *D. melanogaster* when compared to females [29]. In our study, all flies were raised under tightly controlled conditions, suggesting that at least some of the variation we detected among individuals may have had an additive genetic basis. If this is indeed the case, higher temperatures may release cryptic genetic variation in male locomotor activity that may help buffer them against the potentially negative effects of thermal variation. However, coefficients of among-individual variation (mean-standardized variance estimate) differed little between the temperature treatments in either sex, suggesting that the sex-dependent effects of increased temperature on among-individual variation were largely driven by sex differences in average locomotor activity at 28°C. Whether temperature change alters the expression of additive genetic variance differently in males and females and how this is influenced by changes in average locomotor activity will be a key topic for further research. Future experiments that have power to partition genetic variance should repeatedly test the behaviour of individual flies across a broader range of temperatures to investigate whether individual differences in locomotor activity across changing temperatures are heritable. Measuring the survival and reproductive success of these individuals to hone in on associations between temperature-dependent behavioural variation and organismal fitness will be key to understanding and predicting potential sex-specific vulnerabilities to rising temperatures.

Sex differences in within-individual variance in locomotor activity were also linked to temperature; males demonstrated greater within-individual variance with increased temperatures, while females showed the opposite pattern. The findings in males are in line with previous research in aquatic ectotherms, which has similarly found increased within-individual behavioural variance at higher temperatures [17,18,21]. It has been suggested that increased within-individual variation at higher temperatures may be due to the positive effect of temperature on ectothermic metabolism and behavioural activity, where increased temperatures result in a greater amount of energy available to express behavioural variation [10,17]. Indeed, coefficients of within-individual variance in males did not increase substantially at higher temperatures, suggesting that the greater within-individual variance in males at higher temperares was largely driven by their increased average locomotor activity at 28°C. Yet we did not observe such a pattern in our females. On the contrary, we found that within-individual variance in activity rates actually decreased at higher temperatures in females, and this effect was independent of average changes in locomotor activity. This is despite finding that female metabolic rates increased with warmer temperatures, highlighting that increased energy production in response to rising temperatures in ectotherms may not necessarily drive concurrent increases in within-individual behavioural variance.

It is unclear why the effect of temperature on within-individual variation in locomotor activity differed between males and females in our study. We suggest that this effect may again be due to sex differences in energy management strategies, whereby strong positive relationships exist between activity and metabolic rate in males, but not in females [29,30]. Here, increased metabolic rates at higher temperatures may provide males with more energy available to express greater behavioural activity and subsequent within-individual behavioural variation. Conversely, previous research has actually found a negative correlation between evening activity and metabolic rate in female *D. melanogaster*, suggesting a potential energetic trade-off between locomotor performance and metabolism [30]. While locomotor activity was not measured during the evening in the current study, this trade-off between activity and metabolism may partly explain why increased metabolic rates at higher temperatures in females resulted in lower within-individual behavioural variance. While the ecological implications of these sex-differences in within-individual behavioural variability are unclear, we note that previous studies have identified putative associations between within-individual variance and predation in invertebrates [24,57,58]. This suggests that sex-specific effects of temperature on within-individual variation in activity rates found in the current study could lead to temperature-dependent differences between males and females in their vulnerability to predation. Further studies are required to test these links in *D. melanogaster*.

It is also important to highlight that sex differences in residual within-individual variance may have been caused by differences between males and females in measurement error, or sex differences in plasticity in response to unmeasured microenvironmental changes. While we cannot rule this out, we find these explanations for the current results unlikely, given that locomotor activity was automatically tracked using the same equipment for both sexes, and that behavioural trials were conducted at a consistent time of the day in assay chambers with standardized temperature, humidity and lighting. Furthermore, we also note that while not the focus of the current study, we found preliminary evidence for greater among-individual differences in within-individual variance in males when compared to females (table 1). Future quantitative genetic studies will be needed to better understand whether the greater differences between individual males in their within-individual variation has an additive genetic basis and can respond to selection.

In summary, our study revealed key sex differences in thermal behavioural, but not metabolic, plasticity in the vinegar fly. We also found that higher temperatures triggered larger among- and within-individual variation in activity rates in males, but not in females, and that these effects were partly attributable to the influence of higher temperatures on average locomotor activity. Given that increased behavioural variation and a diversity of behavioural strategies have been suggested to enhance population persistence in the face of changing environmental conditions [32,33,59], sex differences in the amount of behavioural variation expressed in response to temperature change may result in sex-specific vulnerability to a warming climate. While our research represents a first step in assessing these implications, future studies investigating whether behavioural differences in thermal responsiveness are heritable and mediate organismal fitness are needed to better understand the adaptive capacity of populations to persist in the face of future climate change.

## Data Availability

Data and statistical code to reproduce the results reported in this manuscript are publically available from the Open Science Framework online repository (https://osf.io/geczs/).
